# The oribatid mite subgenus Galumna (Galumna) (Acari, Oribatida, Galumnidae) in the Philippines

**DOI:** 10.3897/zookeys.452.8212

**Published:** 2014-11-04

**Authors:** Sergey G. Ermilov, Leonila Corpuz-Raros, Andrei V. Tolstikov

**Affiliations:** 1 Tyumen State University, Tyumen, Russia; 2 Crop Protection Cluster, College of Agriculture and Museum of Natural History, University of the Philippines Los Baños, Los Baños, Philippines

**Keywords:** Oribatida, Galumnidae, Galumna (Galumna), new species, supplementary description, key, Philippines

## Abstract

Five species of the subgenus Galumna (Galumna) (Acari, Oribatida, Galumnidae) are registered in the Philippine oribatid mite fauna. A new species, Galumna (Galumna) makilingensis **sp. n.**, is described; it is most similar morphologically to Galumna (Galumna) tokyoensis Aoki, 1966, but differs from the latter by the morphology of porose areas *Aa* and *Ap*, rostral setae, and length of interlamellar setae. Three species, Galumna (Galumna) crenata Deb & Raychaudhuri, 1975, Galumna (Galumna) cf. exigua Sellnick, 1925 and Galumna (Galumna) khoii Mahunka, 1989, are recorded in the Philippines for the first time. The species Galumna (Galumna) crenata is redescribed. An identification key to the Philippine species of Galumna (Galumna) is given.

## Introduction

Galumna (Galumna) is the largest subgenus of *Galumna* Heyden, 1826, comprising 161 species, which have a cosmopolitan distribution (based on data by Subías 2004, updated 2014). In the course of taxonomic identification of oribatid mites from the Philippines, we found five species of this subgenus: one species is represented as a new to science and other four are already known ones (see *Checklist* section below). At present, only Galumna (Galumna) flabellifera Hammer, 1958 was reported from the Philippines (see Corpuz-Raros 1979; Corpuz-Raros and Gruèzo 2011).

The primary goal of the present paper is to describe and illustrate a new species. The secondary goal is to make a supplementary description of Galumna (Galumna) crenata based on the Philippine material, which was originally described by Deb and Raychaudhuri (1975) from India. The first description of Galumna (Galumna) crenata was incomplete, and lacks information on the length of morphological structures, leg setation, solenidia, gnathosoma, and the illustrations were insufficient.

In addition, we present an identification key to the Philippine species of Galumna (Galumna) below.

## Material and methods

The species of Galumna (Galumna) were found in 11 sites:

L-1 Philippines, Mindanao Island, Nasipit Lumber Company, Tungao, Agusan del Norte, in leaf litter, 28.V.1977, collected by J.M. Sotto and R.C. Garcia.

L-3 Philippines, Luzon Island, Animal Science pasture, University of the Philippines Los Baños campus, College, Laguna, in litter from pasture, 28.VI.1975, collected by J.M. Sotto and R.C. Garcia.

L-5 Philippines, Luzon Island, Mt. Makiling, Makiling Botanic Gardens, Los Baños, Laguna, in topsoil from plantation of Moluccan Sau (*Albizia falcataria*), 8.VI.1975, collected by J.M. Sotto and R.C. Garcia.

L-16 Philippines, Luzon Island, Mt. Makiling, Makiling Botanic Gardens, Los Baños, Laguna, in litter from undistrurbed secondary forest, 1.VI.1975, collected by J.M. Sotto and R.C. Garcia.

L-20 Philippines, Luzon Island, Mt. Makiling, Makiling Botanic Gardens, Los Baños, Laguna, in litter from plantation of molave (*Vitex parviflora*), 19.VII.1975, collected by J.M. Sotto and R.C. Garcia.

L-21 Philippines, Luzon Island, Maddela, Quirino, in bamboo leaf litter, 11.XI.1975, collected by P.S. Raros.

L-23 Philippines, Panay Island, Panay State Polytechnic College campus, Mambusao, Capiz, in grass litter, 12.X.1990, collected by A.M. Almeroda.

L-34 Philippines, Luzon Island, Tagga, Tuguegarao, Cagayan, in forest litter, 14.XI.1975, collected by P.S. Raros.

L-40 Philippines, Luzon Island, Animal Science pasture, University of the Philippines Los Baños campus, College, Laguna, in litter at base cogon (*Imperata cylindrica*), 16.IX.1975, collected by J.M. Sotto and R.C. Garcia.

L-43 Philippines, Mindanao Island, Nasipit Lumber Company, Tagpange, Tungao, Agusan del Norte, in litter from *Albizia falcataria*–Ipomoea sp., fern vegetation, 28.IV.1975, collected by R.S. Raros.

L-45 Philippines, Luzon Island, Mt. Makiling, on north trail to peak, Los Baños in litter under pakong-lawit (*Goniophlebium percusum*, Polypodiaceae, fern), 4.V.1975, collected by J.M. Sotto.

Specimens were mounted in lactic acid on temporary cavity slides for measurement and illustration. The body length was measured in lateral view, from the tip of the rostrum to the posterior edge of the ventral fig. The notogastral width refers to the maximum width in dorsal aspect. Lengths of body setae were measured in lateral aspect. All body measurements are presented in micrometers. Formulae for leg setation are given in parentheses according to the sequence trochanter–femur–genu–tibia–tarsus (famulus included). Formulae for leg solenidia are given in square brackets according to the sequence genu–tibia–tarsus. General terminology used in this paper follows that of Grandjean (summarized by Norton and Behan-Pelletier 2009). Drawings were made with the drawing tube using the Carl Zeiss transmission light microscope “Axioskop-2 Plus” at Tyumen State University, Russia.

### Checklist of registered Galumna (Galumna) species

Galumna (Galumna) crenata Deb & Raychaudhuri, 1975. Distribution: India. Locality: L-1. First record in the Philippines.

Galumna (Galumna) cf. exigua Sellnick, 1925. Distribution: Sumatra. Localities: L-1, L-20, L-21, L-23, L-43, L-45. First record in the Philippines.

Galumna (Galumna) flabellifera Hammer, 1958. Distribution: Pantropics and Subtropics. Localities: L-1, L-3, L-5, L-21, L-23, L-34.

Galumna (Galumna) khoii Mahunka, 1989^1^. Distribution: Vietnam. Localities: L-3, L-16, L-23, L-40. First record in the Philippines.

Galumna (Galumna) makilingensis sp. n.: Locality: L-45

## Results

### Description of Galumna (Galumna) makilingensis sp. n.

Taxon classificationAnimaliaOribatidaGalumnidae

http://zoobank.org/4BC74FB7-37C4-4670-8C6D-0BB8BA67C262

Figs 1 , 2 , 3 , 4

#### Diagnosis.

With generic characters of *Galumna* as summarized by Ermilov et al. (2013). Body size: 647–680 × 498–547. Rostrum pointed. Rostral setae of medium size, ciliate. Lamellar and interlamellar setae long, slightly barbed. Bothridial setae spindle-form. Lamellar lines very strong, divergent in distal parts to sublamellar lines. Anterior notogastral margin developed. Four pairs of porose areas present; *Aa* boomerang-like, other rounded or oval. Median pore present. Postanal porose area long, elongated.

#### Description.

*Measurements*. Body length: 647 (holotype, female), 680 (one paratype, female); notogaster width: 498 (holotype), 547 (one paratype).

*Integument*. Body color brown. Body surface smooth.

*Prodorsum*. Rostrum pointed. Rostral setae (*ro*, 49–57) setiform, ciliate unilaterally. Lamellar (*le*, 118–127) and interlamellar (*in*, 172–184) setae setiform, slightly barbed. Bothridial setae (*ss*, 135–147) spindle-form, with long stalk and short, slightly barbed head. Exobothridial setae and their alveoli absent. Porose areas *Ad* oval, transversally oriented (24–32 × 6–10). Sublamellar lines (*S*) distinct, thin, curving backwards. Lamellar lines (*L*) very strong, parallel in basal parts and divergent in distal parts to sublamellar lines.

*Notogaster*. Anterior notogastral margin developed. Dorsophragmata (*D*) of medium size, longitudinally elongated. Notogastral setae represented by 10 pairs of alveoli. Four pairs of porose areas with distinct borders: *Aa* boomerang-like (90–102 × 8–16), other porose areas rounded or oval; *A1* (20–24 × 12–16 or diameter 16–20), *A2* (24–32 × 12–16 or diameter 16–20) and *A3* (24–45 × 20–24). Alveoli *la* inserted posteriorly to *Aa*. Lyrifissures *im* and opisthonotal gland openings (*gla*) located laterally to *A1*. Median pore (*mp*) present, located little posterior to virtual line connecting porose areas *A2*.

*Gnathosoma*. Morphology of subcapitulum, palps and chelicerae generally typical for species of the subgenus Galumna (Galumna) (for example, Engelbrecht 1969; Ermilov and Anichkin 2011; Ermilov et al. 2011). Subcapitulum longer than wide (184 × 155). Subcapitular setae simple, slightly barbed: *a* (32–36) longer than *m* and *h* (both 24–28). Two pairs of adoral setae (20) setiform, barbed. Palps (135–139) with setation 0–2–1–3–9(+ω). Solenidion straight, thickened, blunt-ended, attached to eupathidium. Chelicerae (229) with two setiform, barbed setae; *cha* (57) longer than *chb* (32). Trägårdh’s organ distinct, tapered.

*Epimeral and lateral podosomal regions*. Apodemes (1, 2, sejugal, 3) well visible. Four pairs of setiform, slightly barbed epimeral setae present; *1a* and *3b* (41–49) longer than *4a* and *4b* (24–32). Pedotecta II rectangular, rounded anteriorly in ventral view. Discidia (*dis*) rounded distally. Circumpedal carinae (*cp*) of medium length, directed to *3b*.

*Anogenital region*. Six pairs of genital (*g*_1_–*g*_6_, 20–24), one pair of aggenital (*ag*, 20–24), two pairs of anal (*an*_1_, *an*_2_, 28–32) and three pairs of adanal (*ad*_1_–*ad*_3_, 28–32) setae setiform, slightly barbed. Anal and adanal setae slightly thicker than genital and aggenital setae. Anterior edge of genital figs with two setae. Adanal setae *ad*_3_ inserted laterally to adanal lyrifissures *iad*. Postanal porose area (*Ap*) long, elongated, transversally oriented (61–77 × 8–12).

*Legs*. Morphology of leg segments, setae and solenidia generally typical for species of the subgenus Galumna (Galumna) (for example, Engelbrecht 1969; Ermilov and Anichkin 2011; Ermilov et al. 2011; Bayartogtokh and Akrami 2014). Formulae of leg setation and solenidia: I (1–4–3–4–20) [1–2–2], II (1–4–3–4–15) [1–1–2], III (1–2–1–3–15) [1–1–0], IV (1–2–2–3–12) [0–1–0]; homology of setae and solenidia indicated in Table 1.

**Figure 1.**
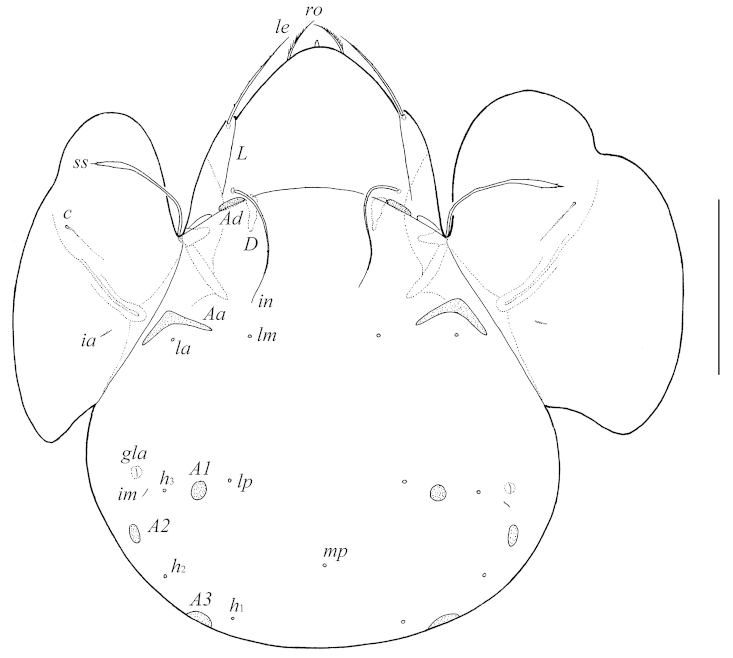
Galumna (Galumna) makilingensis sp. n., adult: dorsal view. Scale bar 200 μm.

**Figure 2.**
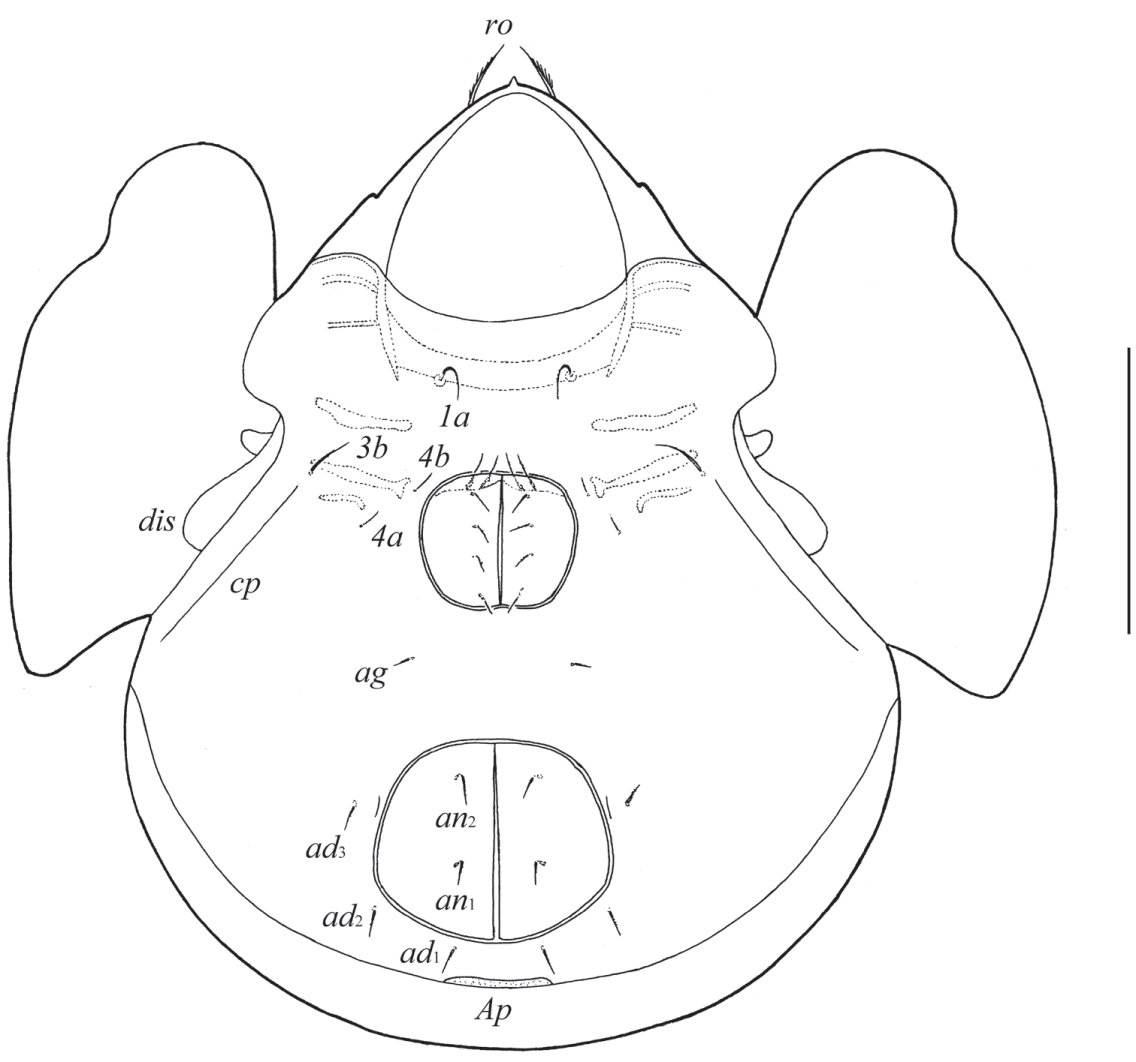
Galumna (Galumna) makilingensis sp. n., adult: ventral view (gnathosoma and legs not illustrated). Scale bar 200 μm.

**Figures 3–4.**
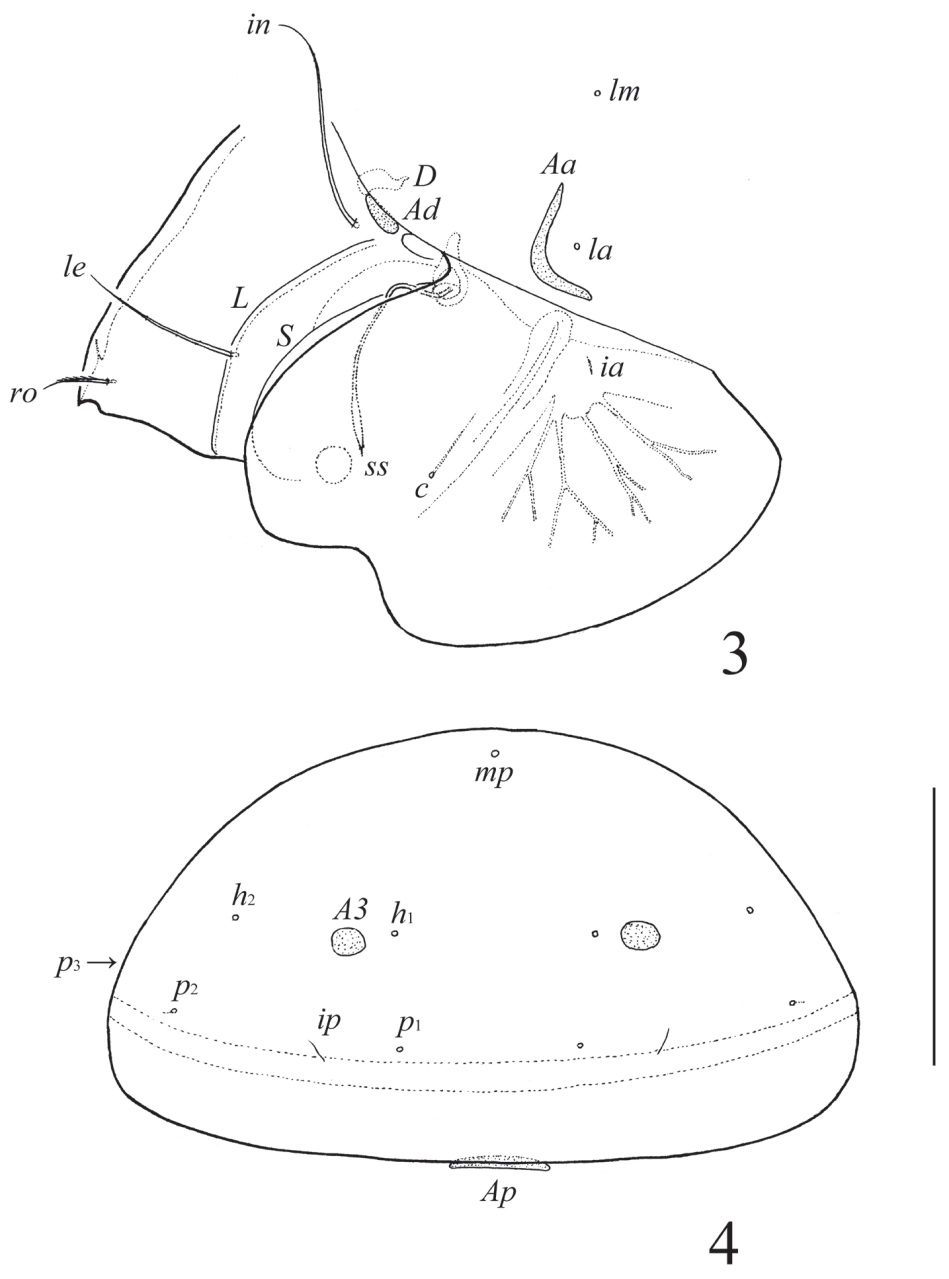
Galumna (Galumna) makilingensis sp. n., adult: **3** dorso-lateral view of prodorsum, left pteromorph and anterior part of notogaster (gnathosoma and leg I not illustrated) **4** posterior view of notogaster. Scale bar 200 μm.

**Table 1.** Leg setation and solenidia of Galumna (Galumna) makilingensis sp. n. (same data for Galumna (Galumna) crenata Deb & Raychaudhuri, 1975).

Leg	Trochanter	Femur	Genu	Tibia	Tarsus
I	*v*’	*d*, *(l)*, *bv*’’	*(l)*, *v*’,﻿ σ	*(l)*, *(v)*,﻿ φ_1_, φ_2_	*(ft)*, *(tc)*, *(it)*, *(p)*, *(u)*, *(a)*, *s*, *(pv)*, *v*’, *(pl)*, *l*’’, ﻿ε,﻿ ω_1_, ω_2_
II	*v*’	*d*, *(l)*, *bv*’’	*(l)*, *v*’,﻿ σ	*(l)*, *(v)*,﻿ φ	*(ft)*, *(tc)*, *(it)*, *(p)*, *(u)*, *(a)*, *s*, *(pv)*,﻿ ω_1_, ω_2_
III	*v*’	*d*, *ev*’	*l*’,﻿ σ	*l*’, *(v)*,﻿ φ	*(ft)*, *(tc)*, *(it)*, *(p)*, *(u)*, *(a)*, *s*, *(pv)*
IV	*v*’	*d*, *ev*’	*d*, *l*’	*l*’, *(v)*,﻿ φ	*ft*’’, *(tc)*, *(p)*, *(u)*, *(a)*, *s*, *(pv)*

Roman letters refer to normal setae (ε to famulus), Greek letters to solenidia. Single prime (‘) marks setae on anterior and double prime (“) setae on posterior side of the given leg segment. Parentheses refer to a pair of setae.

#### Material examined.

Holotype (female) and one paratype (female): L-45.

#### Type deposition.

The holotype is deposited in the collection of the Zoological Institute of the Russian Academy of Sciences, St. Petersburg, Russia; one paratype (dissected) is deposited in the collection of the Tyumen State University Museum of Zoology, Tyumen, Russia.

#### Etymology.

The specific name “*makilingensis*” refers to the type locality, Mt. Makiling, the forest reservation of the University of the Philippines Los Baños.

#### Comparison.

In having large body size, pointed rostrum, spindle-form bothridial setae, long prodorsal setae, anterior notogastral margin, four pairs of notogastral porose areas, Galumna (Galumna) makilingensis sp. n. is most similar to Galumna (Galumna) tokyoensis Aoki, 1966 from the Palaearctic region (Aoki 1966). However, it clearly differs from the latter by the boomerang-like porose areas *Aa* and long, elongated postanal porose area (versus both oval in Galumna (Galumna) tokyoensis), ciliate rostral setae (versus smooth in Galumna (Galumna) tokyoensis) and interlamellar setae longer than lamellar setae (versus similar in length in Galumna (Galumna) tokyoensis).

Also, in having large body size, bothridial setae with dilated head, long prodorsal setae, anterior notogastral margin, four pairs of notogastral porose areas, *Aa* boomerang-like, Galumna (Galumna) makilingensis sp. n. is most similar to Galumna (Galumna) cuneata Aoki, 1961 from the Palaearctic region (Aoki 1961). However, it clearly differs from the latter by the pointed rostrum (versus rounded in Galumna (Galumna) cuneata), ciliate rostral setae (versus smooth in Galumna (Galumna) cuneata), interlamellar setae longer than lamellar setae (versus similar in length in Galumna (Galumna) cuneata) and spindle-form bothridial setae (versus clavate in Galumna (Galumna) cuneata).

### Supplementary description of Galumna (Galumna) crenata

Taxon classificationAnimaliaOribatidaGalumnidae

Deb & Raychaudhuri, 1975

Figs 5 , 6 , 7 , 8

#### Description.

*Measurements*. Body length: 348–390 (four specimens, two females and two males); notogaster width: 258–290 (four specimens).

*Integument*. Body color brown. Body surface smooth, but ventral side covered by the microgranular cerotegument (diameter of granules less than 1), visible only under high magnification (×1000) in dissected specimens. Genital figs with one longitudinal stria in medial parts.

*Prodorsum*. Rostrum rounded. Rostral setae (24–32) setiform, smooth. Lamellar and interlamellar setae minute (both 6–8), thin, smooth. Bothridial setae (49–57) clavate, with long stalk and shorter, rounded and weakly barbed distally head. Exobothridial setae and their alveoli absent. Porose areas *Ad* large, oval, transversally oriented (20–22 × 6–8). Lamellar and sublamellar lines distinct, thin, parallel, curving backwards.

*Notogaster*. Anterior notogastral margin developed, but sometimes poorly visible. Dorsophragmata of medium size, longitudinally elongated. Notogastral setae represented by 10 pairs of alveoli. Four pairs of porose areas with distinct borders: *Aa* large, boot-shaped or weakly triangular, transversally oriented (32–36 × 12–16); *A1*, *A2* (diameter of both 8–16) and *A3* (diameter of 14–20) rounded. Alveoli *la* inserted posteriorly to *Aa*. Lyrifissures *im* located between *lm* and *lp*. Opisthonotal gland openings located laterally to *A1*. Median pore present, located little anterior to virtual line connecting porose areas *A3*.

*Gnathosoma*. Morphology of subcapitulum, palps and chelicerae generally typical for species of the subgenus Galumna (Galumna) (for example, Engelbrecht 1969; Ermilov and Anichkin 2011; Ermilov et al. 2011). Subcapitulum longer than wide (90–94 × 82–68). Subcapitular setae simple, smooth: *a* (14–16) longer and thicker than *m* and *h* (both 6–8). Two pairs of adoral setae (8) setiform, slightly barbed. Palps (57) with setation 0–2–1–3–9(+ω). Solenidion straight, thickened, blunt-ended, attached to eupathidium. Chelicerae (106–110) with two setiform, barbed setae; *cha* (36) longer than *chb* (20). Trägårdh’s organ distinct, tapered.

*Epimeral and lateral podosomal regions*. Anterior tectum of epimere I with numerous rectangular teeth. Apodemes (1, 2, sejugal, 3) well visible. Six pairs of thin, smooth epimeral setae (8–12) present. Setae *4c* inserted on tubercle. Pedotecta II rectangular, rounded anteriorly in ventral view. Discidia triangular. Circumpedal carinae long, directed to pedotecta I.

*Anogenital region*. Six pairs of genital setae (*g*_1_, *g*_2_, 8, *g*_3_–*g*_6_, 4–6), one pair of aggenital (*ag*, 6–8), two pairs of anal (*an*_1_, *an*_2_, 8) and three pairs of adanal (*ad*_1_–*ad*_3_, 8) setae thin, smooth. Anterior edge of genital figs with two setae. Adanal setae *ad*_3_ inserted laterally to adanal lyrifissures *iad*. Postanal porose area oval, transversally oriented (12–20 × 6–8).

*Legs*. Morphology of leg segments, setae and solenidia generally typical for species of the subgenus Galumna (Galumna) (for example, Engelbrecht 1969; Ermilov and Anichkin 2011; Ermilov et al. 2011; Bayartogtokh and Akrami 2014). Formulae of leg setation and solenidia: I (1–4–3–4–20) [1–2–2], II (1–4–3–4–15) [1–1–2], III (1–2–1–3–15) [1–1–0], IV (1–2–2–3–12) [0–1–0]; homology of setae and solenidia indicated in Table 1.

**Figure 5.**
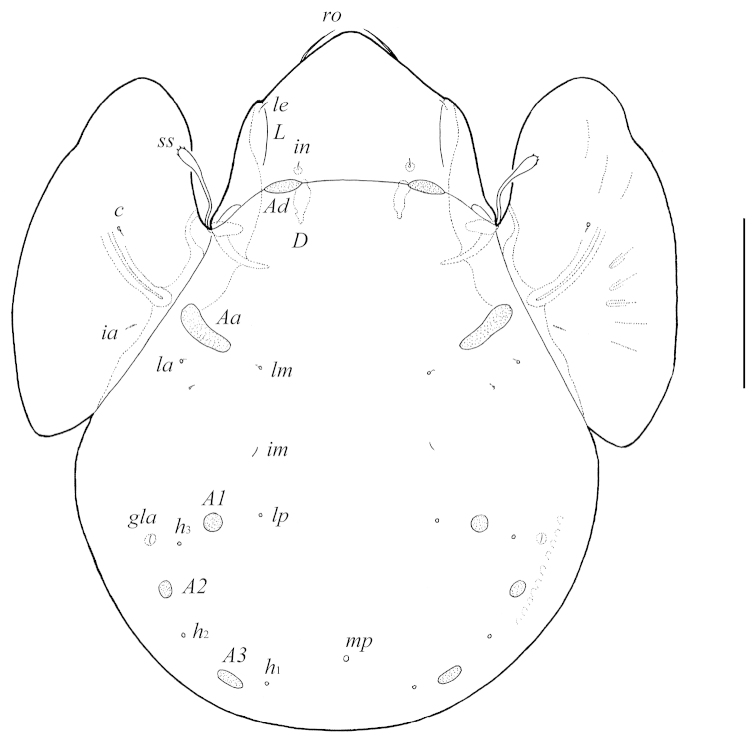
Galumna (Galumna) crenata Deb & Raychaudhuri, 1975, adult: dorsal view. Scale bar 100 μm.

**Figure 6.**
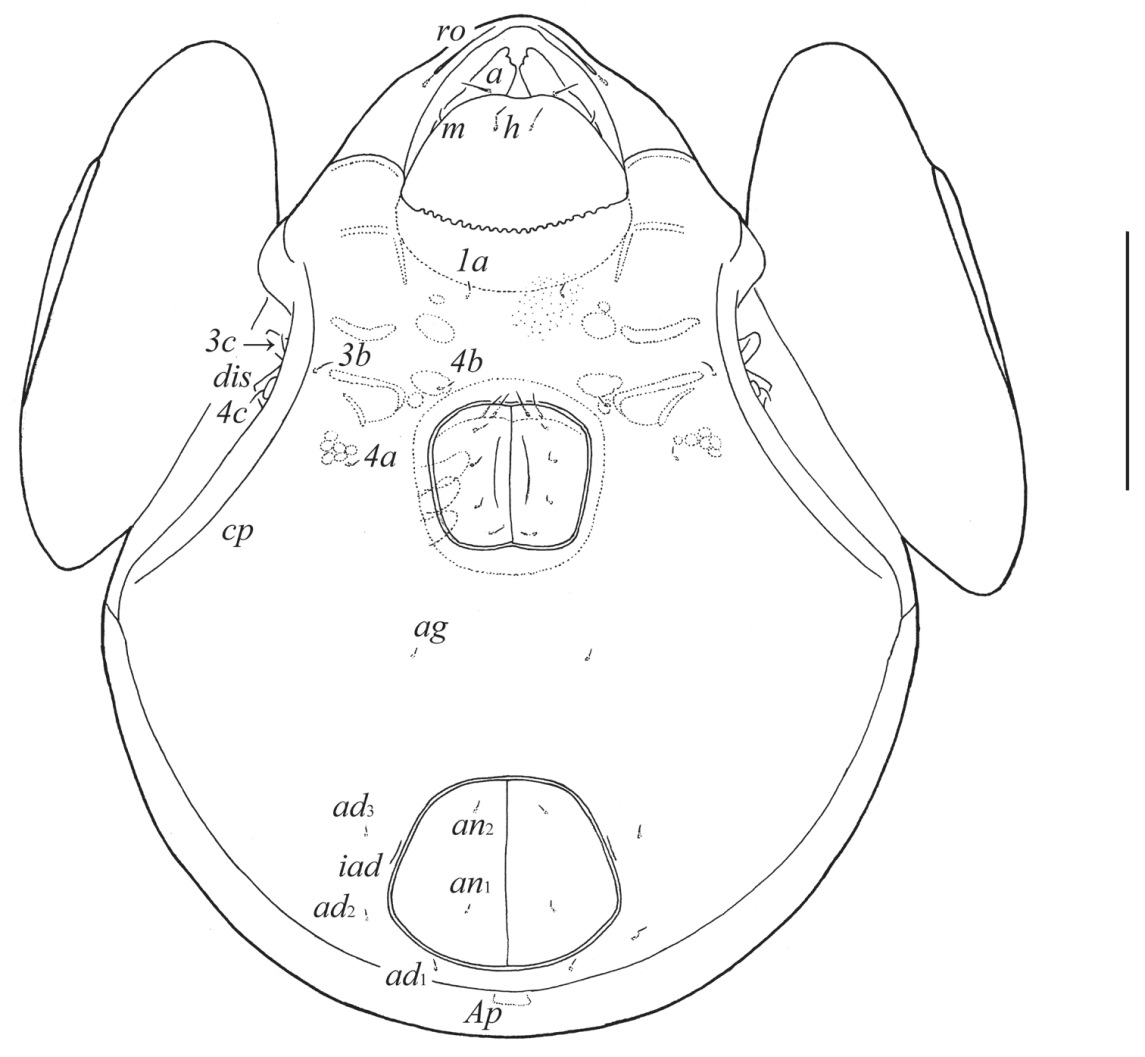
Galumna (Galumna) crenata Deb & Raychaudhuri, 1975, adult: ventral view (legs not illustrated). Scale bar 100 μm.

**Figures 7–8.**
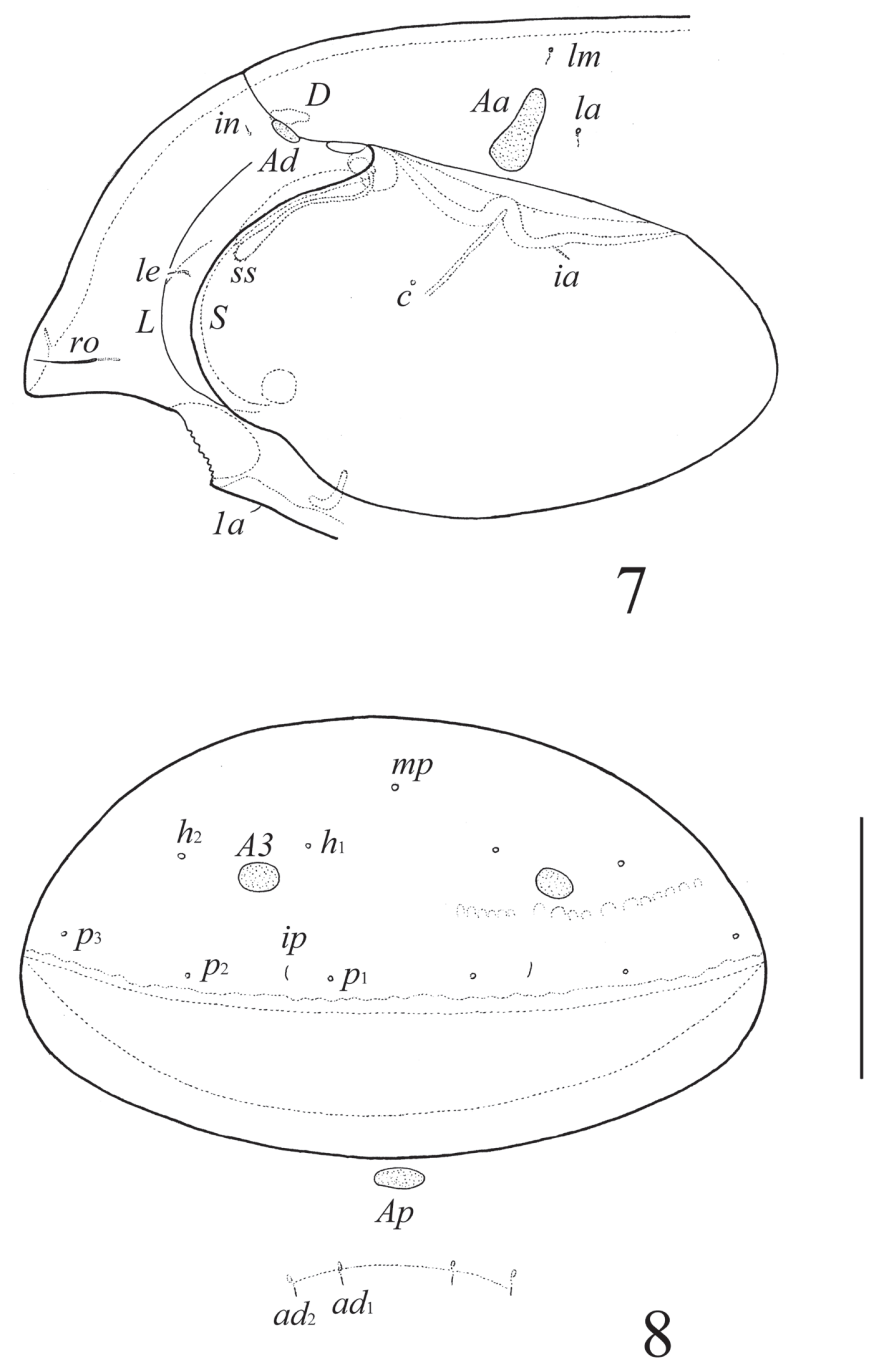
Galumna (Galumna) crenata Deb & Raychaudhuri, 1975, adult: **7** lateral view of prodorsum, left pteromorph and anterior part of notogaster (gnathosoma and leg I not illustrated) **8** posterior view of notogaster and adanal setae *ad*_1_ and *ad*_2_. Scale bar 100 μm.

#### Material examined.

Four specimens (two females and two males): L-1.

#### Remarks.

Galumna (Galumna) crenata distinctly differs from other species of the sugenus by the presence of dentate anterior tectum of epimere I. The available Philippine specimens of this species are morphologically and in general appearance similar to the Indian specimens (Deb and Raychaudhuri, 1975). Three main differences are as follows:

1. Body longer (348–390 versus 319–325 in Indian specimens). We believe these differences represent intraspecific (perhaps geographical) variability.
2. Anterior notogastral margin is well visible (versus completely absent in Indian specimens); also, the text of other paper (Sarkar et al. 2007) on Galumna (Galumna) crenata assert that it is present.
3. Rostral, lamellar and interlamellar setae developed (versus absent in Indian specimens). We believe these differences can be erroneous. The reason is that Deb and Raychaudhuri (1975) inadequately described this species and, probably, they overlooked these setae, because the rostral setae are usually strongly pressed to the prodorsum surface and are often not visible in dorsal and ventral views, and the lamellar and interlamellar setae are minute, well visible only under high magnification.

### Key to species of Galumna (Galumna) of the Philippines

**Table:** 

1	Rostrum pointed; porose areas *Aa* boomerang-like; body size: 647–680 × 498–547	**Galumna (Galumna) makilingensis sp. n.** Distribution: Philippines.
–	Rostrum rounded; porose areas *Aa* not boomerang-like	**2**
2	Lamellar and interlamellar setae well developed, long; bothridial setae lanceolate; body size: 425–482 × 305–344	**Galumna (Galumna) khoii Mahunka, 1989**. Distribution: Vietnam and Pilippines.
–	Lamellar and interlamellar setae minute or absent; bothridial setae clavate	**3**
3	Porose areas *Aa* elongated, transversally oriented, boot-shaped or weakly triangular; anterior tectum of epimere I dentate; body size: 319–390 × 249–290	**Galumna (Galumna) crenata Deb & Raychaudhuri, 1975** (including our data). Distribution: India and Philippines.
–	Porose areas *Aa* rounded; anterior tectum of epimere I smooth	**4**
4	Bothridial heads densely ciliale; anterior notogastral margin developed; body size: 303–348 × 204–220	**Galumna (Galumna) flabellifera Hammer, 1958** (see also Aoki 1964, 1965, 1982). Distribution: Pantropics and Subtropics.
–	Bothridial head smooth; anterior notogastral margin not developed; body size: 330 × 264	**Galumna (Galumna) cf. exigua Sellnick, 1925.** Distribution: Sumatra and Philippines.

## Supplementary Material

XML Treatment for Galumna (Galumna) makilingensis

XML Treatment for Galumna (Galumna) crenata

## Footnotes

1 It is possible that Galumna (Galumna) khoii Mahunka, 1989 is a junior synonym of Galumna (Galumna) lanceata (Oudemans, 1900) (see Ermilov and Anichkin 2014).
